# Polyphasic characterisation of *Burkholderia cepacia* complex species isolated from children with cystic fibrosis

**DOI:** 10.1590/0074-02760150314

**Published:** 2016-01

**Authors:** Fernando José Vicenzi, Marcelo Pillonetto, Helena Aguilar Peres Homem de Mello de Souza, Jussara Kasuko Palmeiro, Carlos Antônio Riedi, Nelson Augusto Rosario-Filho, Libera Maria Dalla-Costa

**Affiliations:** 1 Laboratório Municipal de Curitiba, Seção de Imunoquímica, Curitiba, PR, Brasil; 2 Faculdades Pequeno Príncipe, Instituto de Pesquisa Pelé Pequeno Principe, Curitiba, PR, Brasil; 3 Pontifícia Universidade Católica do Paraná, Escola de Saúde e Biociências, Curso de Farmácia, Departamento de Microbiologia, Curitiba, PR, Brasil; 4 Laboratório Central do Estado do Paraná, Seção de Biologia Molecular, Curitiba, PR, Brasil; 5 Universidade Federal do Paraná, Faculdade de Medicina, Hospital de Clínicas, Seção de Bacteriologia, Curitiba, PR, Brasil; 6 Universidade Federal do Paraná, Faculdade de Medicina, Departamento de Pediatria, Curitiba, PR, Brasil

**Keywords:** *Burkholderia cepacia* complex, cystic fibrosis, lung disease, Gram-negative bacterial infection, DNA sequence analyses, matrix-assisted laser desorption-ionisation mass spectrometry

## Abstract

Cystic fibrosis (CF) patients with *Burkholderia cepacia* complex (Bcc) pulmonary infections have high morbidity and mortality. The aim of this study was to compare different methods for identification of Bcc species isolated from paediatric CF patients. Oropharyngeal swabs from children with CF were used to obtain isolates of Bcc samples to evaluate six different tests for strain identification. Conventional (CPT) and automatised (APT) phenotypic tests, polymerase chain reaction (PCR)-*recA*, restriction fragment length polymorphism-*recA*, *recA*sequencing, and matrix-assisted laser desorption ionization-time of flight (MALDI-TOF) were applied. Bacterial isolates were also tested for antimicrobial susceptibility. PCR-*recA* analysis showed that 36 out of the 54 isolates were Bcc. Kappa index data indicated almost perfect agreement between CPT and APT, CPT and PCR-*recA*, and APT and PCR-*recA* to identify Bcc, and MALDI-TOF and *recA*sequencing to identify Bcc species. The *recA*sequencing data and the MALDI-TOF data agreed in 97.2% of the isolates. Based on *recA* sequencing, the most common species identified were *Burkholderia cenocepacia* IIIA (33.4%),*Burkholderia vietnamiensis* (30.6%), *B. cenocepacia*IIIB (27.8%), *Burkholderia multivorans* (5.5%), and *B. cepacia* (2.7%). MALDI-TOF proved to be a useful tool for identification of Bcc species obtained from CF patients, although it was not able to identify *B. cenocepacia* subtypes.

Cystic fibrosis (CF) pathophysiology leads to an increased susceptibility to respiratory infections (Drevinek & Mahenthiralingam 2010). The *Burkholderia cepacia* complex (Bcc) prevalence rates in CF patients vary geographically and have been shown to be 1.8% in Belgium (De Boeck et al. 2004) and 5% in Canada (CFC 2015); in Brazilian children, the prevalence was 4% (Souza et al. 2006). CF patients with Bcc infection have high rates of morbidity and mortality (Drevinek & Mahenthiralingam 2010, LiPuma 2010). Bcc infections pose potential for patient-to-patient transmission, which can lead to the development of “cepacia syndrome,” a fatal necrotising pneumonia (Drevinek & Mahenthiralingam 2010).

Currently, Bcc includes 20 species; however, new species have been identified or reclassified over the years (Drevinek & Mahenthiralingam 2010, LiPuma 2010,Vandamme & Dawyndt 2011, Peeters et al. 2013, Smet et al. 2015). *Burkholderia multivorans* and*Burkhol- deria cenocepacia* were shown to be the prevalent species (Drevinek & Mahenthiralingam 2010, LiPuma 2010). Correct species identification of Bcc is essential for treating CF patients (Gilligan et al. 2006) because of interspecies variation in antimicrobial susceptibility (Leitão et al. 2008) and potential for patient-to-patient transmission (Drevinek & Mahenthiralingam 2010). However, accurate Bcc identification is challenging due to the phenotypic and genetic similarities among species.

Bcc species comprise phenotypically indistinguishable microorganisms with high genetic similarity (> 97.5%) that was determined using 16S rDNA sequencing (Coenye et al. 2001, Vandamme & Dawyndt 2011). Conversely, 16S rDNA sequencing assays cannot accurately discriminate among *B. cepacia*, *B. cenocepacia*, *Burkholderia ambifaria*, *Burkholderia pyrrocinia*, and *Burkholderia anthina* (Vermis et al. 2002b). Furthermore, *recA* sequencing data showed that the Bcc interspecies and intraspecies similarities were 94-95% and 98-99%, respectively (Vanlaere et al. 2008, Vandamme & Dawyndt 2011). Thus, Bcc inter and intraspecies discrimination is challenging.

The discriminatory power of *recA* sequencing method is better than 16S rDNA sequencing analysis because *recA* has enough nucleotide variability to enable differentiation of the main Bcc species isolated from CF patients (Mahenthiralingam et al. 2000).

Selective media culture and several methods, including phenotypic methods, conventional and automated methods, have been used for Bcc identification. However, reliable and accurate identification of Bcc requires the use of molecular assays such as polymerase chain reaction (PCR)-based assays, PCR-restriction fragment length polymorphism (RFLP-PCR), DNA sequencing, and matrix-assisted laser desorption ionization-time of flight (MALDI-TOF). A study comparing the proficiency of these tests would be valuable for developing a multi-method approach with higher accuracy.

The aim of this study was to compare different methods for identification of Bcc species isolated from oropharyngeal swabs of paediatric CF patients.

## SUBJECTS, MATERIALS AND METHODS

*Bacterial samples* - Oropharyngeal swabs were collected from 46 CF patients (aged 2-90 months) attended at the Cystic Fibrosis Outpatient Clinic of the Department of Paediatrics of the Federal University of Paraná (UFPR) Clinics Hospital from August 2003-February 2009 (Souza et al. 2006, Souza 2012) as previously described (Souza et al. 2006).

Fifty-four bacteria samples grown in *B. cepacia* selective agar (Henry et al. 1997), presumptively identified as Bcc by polymyxin B susceptibility, oxidase test, and lysine decarboxylation test, were included in the study. Bacteria isolates were kept at -80ºC until subjected to the methods studied.

*Phenotypic identification* - Fifty-four bacterial isolates were subjected to conventional phenotypic tests (CPT) and automated phenotypic tests (APT). CPT was performed as previously described (Schreckenberger et al. 2003). APT identification was carried out using GN ID cards in the Vitek^®^ 2 Compact system (bioMérieux, USA).

*Susceptibility testing* - Minimum inhibitory concentrations (MICs) of ceftazidime (CAZ), meropenem (MER), levofloxacin (LEV), and sulfamethoxazole/trimethoprim (SUT) were performed (in 36 bacterial isolates) using the agar dilution test protocol described in Clinical and Laboratory Standards Institute (CLSI) M07-A09 (CLSI 2012) and by using interpretive criteria described in CLSI M100-S24 (CLSI 2014). *Pseudomonas aeruginosa* ATCC 27853 and*Escherichia coli* ATCC 25922 were used as control strains.

*Extraction, purification, and quantification of bacterial DNA* - Template DNA of the 54 bacterial isolates was obtained using post-boiling extraction technique (Navrátilová et al. 2013) for performing *recA* PCR amplification and RFLP-PCR (Mahenthiralingam et al. 2000). AccuPrep Genomic DNA Extraction kit (Bioneer Corporation, Korea) were used for the 36 bacterial isolates subject to *recA* sequencing. The DNA concentrations of samples were quantified using Nano Vue Plus (GE Healthcare Bio-Sciences Corp, USA) and adjusted to concentrations between 20-50 ng/µL.

*PCR for recA* - Primers BCR1 (5′→3′TGAGCCGCCGCAAGAAGAA) and BCR2 (5′→3′CTCTTCCATTTCGTCCTCCGC) (Mahenthiralingam et al. 2000) were used for the identification of Bcc. PCR mixture (50 µL) consisted of 1.5 mM MgCl_2_, 0.4 µM of each primer, 0.2 mM each deoxynucleoside triphosphate, 0.02 U/µL Taq DNA polymerase, 1× PCR buffer, 5 µL template DNA (20-50 ng/µL), and 35.5 µL of PCR-grade water. Amplification was performed using Techne TC-412 thermal cycler (Bibby Scientific Ltd, UK) under the following conditions: 1 cycle at 95ºC for 5 min followed by 35 cycles at 95ºC for 30 s, 65ºC for 45 s, and 72ºC for 60 s. The final extension was performed using 1 cycle at 72ºC for 10 min. *B. cepacia* ATCC 17759 and *P. aeru- ginosa* ATCC 27853 were used as positive and negative controls, respectively. The Bcc-positive were confirmed by the presence of a band of approximately 1,040 bp.

*PCR-RFLP for recA* - Amplicons obtained with BCR1 and BCR2 primers were digested with *Hae*III enzyme and each agarose gel (1.5%) electrophoresis profile was compared to those of the following control strains:*B. cenocepacia* IIIA, *B. cenocepacia* IIIB,*Burkholderia stabilis*, *B. multivorans*,*B. cepacia*, and *Burkholderia vietnamiensis* to identify the Bcc species (Mahenthiralingam et al. 2000).

*recA sequencing* - The *recA* amplicons were purified with ExoSAP-IT (Affymetrix, USA) and then sequenced using BigDye Terminator v.3.1 Cycle Sequencing Kit (Applied Biosystems, USA). The 1,043 bp *recA*gene was sequenced in two fragments of approximately 500 bp using a combination of primers BCR1 (5′→3′TGAGCCGCCGCAAGAAGAA) with BCR4 (5′→3′GCGCAGCGCCTGCGACAT) and BCR2 (5′→3′CTCTTCCATTTCGTCCGCCTC) with BCR3 (5′→3′GTCGCAGGCGCTGCGCAA) (Mahenthiralingam et al. 2000). The amplicons were purified using BigDye sequencing X Terminator Purification Kit (Applied Biosystems, Life Technologies Corporation) and sequenced. Each sample generated four sequences that were analysed by using Bioedit software (mbio.ncsu.edu/bioedit/bioedit.html). The obtained consensus sequence was then submitted to PubMLST Bcc allele *recA* database (pubmlst.org/bcc/).

*MALDI-TOF analysis* - MALDI-TOF protein extraction was performed as previously described (Khot et al. 2012). The extract was inoculated in duplicate, air-dried, and covered with 1 mL saturated α-cyano-4-hydroxycinnamic acid in 50% acetonitrile and 2.5% trifluoroacetic acid solution. Mass spectra were determined by using Bruker Daltonics Microflex LT instrument with MALDI Biotyper 3.0 software (Bruker Daltonics, Germany), previously calibrated with Bruker Bacterial Test Standard (*Escherichia coli*DH5α) and the mass spectra were generated using a mass range of 2–kDa obtained by ionisation of 240 shots. Species identification and genus were obtained when the scores were ≥ 2.0 and between ≥ 1.7 and < 2.0, respectively, as recommended by the manufacturer. *B. cepacia* ATCC 25608, *P. aeruginosa* ATCC 27853, and *E. coli* ATCC 25922 were used as controls.

*Statistical analyses* - The analysis of agreement between the methods was performed using the kappa (k) test with criteria outlined by Landys & Koch (1977): poor agreement (k < 0), slight agreement (k: 0.01-0.2), reasonable agreement (k: 0.21-0.40), moderate agreement (k: 0.41-0.60), substantial agreement (k: 0.61-0.80), and almost perfect agreement (k: 0.81-1.00). APT and CPT proficiency analyses were performed using contingency table or 2×2 matrix.

*Ethics* - The study design and the consent form were approved by the Clinics Hospital/UFPR Institutional Ethical Review Board.

## RESULTS

Thirty-six out of the fifty-four preliminarily identified Bcc strains were confirmed as Bcc by using *recA* PCR amplification. The discordancy in Bcc identification was highlighted by comparative analysis of the findings of the five methodologies (CPT, APT, *recA* PCR, *recA*sequencing, and MALDI-TOF) (Table I). Notably, identification of one bacterial isolate did not agree by different techniques: *Bordetella* spp by CPT, *Cupriavidus pauculus* by APT, and as *B. cepacia* by both*recA* sequencing method and MALDI-TOF. One strain of *B. vietnamiensis* could not be conclusively identified by using CPT (Table I).

**TABLE I:** *Burkholderia cepacia* complex (Bcc) identification data obtained by using different methodologies

Patient	Isolate	CPT	APT	PCR *recA*	Seq.RecA PubMLST Bcc database	MALDI-TOF	Allele *recA*	RFLP
1	BC-48-FC	*Bordetella* sp.	*Cupriavidus pauculus*	Bcc	*B. cenocepacia* IIIB	*B. cenocepacia*	15	F
	BC-5-FC	Bcc	Bcc	Bcc	*B. cepacia*	*B. cepacia*	37	E
2	BC-28-FC	Bcc	Bcc	Bcc	*B. cenocepacia* IIIB	*B. cenocepacia*	15^*a*^	F
	BC-54-FC	Bcc	Bcc	Bcc	*B. cenocepacia* IIIB	*B. cenocepacia*	15^*a*^	F
3	BC-25-FC	Bcc	Bcc	Bcc	*B. cenocepacia* IIIA	*B. cenocepacia*	14^*a*^	A
4	BC-32-FC	Bcc	Bcc	Bcc	*B. cenocepacia* IIIA	*B. cenocepacia*	14^*a*^	A
5	BC-46-FC	Bcc	Bcc	Bcc	*B. cenocepacia* IIIB	*B. cenocepacia*	15^*a*^	C
6	BC-10-FC	Bcc	Bcc	Bcc	*B. cenocepacia* IIIA	*B. cenocepacia*	14	A
	BC-15-FC	Bcc	Bcc	Bcc	*B. cenocepacia* IIIA	*B. cenocepacia*	14^*a*^	A
7	BC-1-FC	Bcc	Bcc	Bcc	*B. multivorans*	*B. multivorans*	81^*a*^	D
	BC-20-FC	Bcc	Bcc	Bcc	*B. multivorans*	*B. multivorans*	81^*a*^	D
8	BC-11-FC	Bcc	Bcc	Bcc	*B. vietnamiensis*	*B. cenocepacia*	23^*a*^	H
	BC-18-FC	Bcc	Bcc	Bcc	*B. vietnamiensis*	*B. vietnamiensis*	23^*a*^	H
	BC-19-FC	Bcc	Bcc	Bcc	*B. vietnamiensis*	*B. vietnamiensis*	23	H
	BC-21-FC	Bcc	Bcc	Bcc	*B. vietnamiensis*	*B. vietnamiensis*	23^*a*^	H
	BC-29-FC	Bcc	Bcc	Bcc	*B. vietnamiensis*	*B. vietnamiensis*	23^*a*^	H
	BC-42-FC	Inconclusive	Bcc	Bcc	*B. vietnamiensis*	*B. vietnamiensis*	23^*a*^	H
	BC-58-FC	Bcc	Bcc	Bcc	*B. vietnamiensis*	*B. vietnamiensis*	23^*a*^	H
	BC-59-FC	Bcc	Bcc	Bcc	*B. vietnamiensis*	*B. vietnamiensis*	23^*a*^	H
	BC-7-FC	Bcc	Bcc	Bcc	*B. vietnamiensis*	*B. vietnamiensis*	23^*a*^	H
9	BC-27-FC	Bcc	Bcc	Bcc	*B. cenocepacia* IIIB	*B. cenocepacia*	15^*a*^	F
10	BC-33-FC	Bcc	Bcc	Bcc	*B. cenocepacia* IIIA	*B. cenocepacia*	14^*a*^	A
	BC-3-FC	Bcc	Bcc	Bcc	*B. cenocepacia* IIIA	*B. cenocepacia*	14^*a*^	A
11	BC-36-FC	Bcc	Bcc	Bcc	*B. cenocepacia* IIIA	*B. cenocepacia*	14^*a*^	A
	BC-56-FC	Bcc	Bcc	Bcc	*B. cenocepacia* IIIB	*B. cenocepacia*	15^*a*^	C
	BC-60-FC	Bcc	Bcc	Bcc	*B. cenocepacia* IIIB	*B. cenocepacia*	15^*a*^	C
	BC-61-FC	Bcc	Bcc	Bcc	*B. cenocepacia* IIIB	*B. cenocepacia*	122	C
12	BC-2-FC	Bcc	Bcc	Bcc	*B. vietnamiensis*	*B. vietnamiensis*	48^*a*^	I
13	BC-50-FC	Bcc	Bcc	Bcc	*B. vietnamiensis*	*B. vietnamiensis*	48^*a*^	I
14	BC-22-FC	Bcc	Bcc	Bcc	*B. cenocepacia* IIIB	*B. cenocepacia*	15^*a*^	F
15	BC-34-FC	Bcc	Bcc	Bcc	*B. cenocepacia* IIIA	*B. cenocepacia*	14^*a*^	A
	BC-38-FC	Bcc	Bcc	Bcc	*B. cenocepacia* IIIA	*B. cenocepacia*	14^*a*^	A
	BC-39-FC	Bcc	Bcc	Bcc	*B. cenocepacia* IIIA	*B. cenocepacia*	14^*a*^	A
	BC-41-FC	Bcc	Bcc	Bcc	*B. cenocepacia* IIIA	*B. cenocepacia*	14^*a*^	A
	BC-57-FC	Bcc	Bcc	Bcc	*B. cenocepacia* IIIA	*B. cenocepacia*	14^*a*^	A
16	BC-52-FC	Bcc	Bcc	Bcc	*B. cenocepacia* IIIB	*B. cenocepacia*	49^*a*^	B

*a*: exact *recA* allele match; APT: automated phenotypic tests; CPT: conventional phenotypic tests; MALDI-TOF: matrix-assisted laser desorption ionization-time of flight; PCR: polymerase chain reaction; RFLP: restriction fragment length polymorphism.

The *recA* sequencing data and MALDI-TOF data, for the majority of the 36 strains, were in agreement and corroborated with Bcc identification data. However, one strain identified as *B. vietnamiensis* by*recA* sequencing was identified as *B. cenocepacia* by MALDI-TOF (Table I).

The identification data obtained by the aforementioned methods were used for performing comparative analyses to assess whether Bcc or Bcc genomic variants were identified consistently. The concordance between phenotypic test data (CPT vs. APT) and that among different tests was also assessed (Table II).

**TABLE II:** Evaluation of agreement among methods

Tests (n)^*a*^	k	SE	Agreement^*b*^
CPT vs. APT (54)	0.833	0.080	Almost perfect agreement
CPT vs. *recA* PCR^*c*^(54)	0.833	0.080	Almost perfect agreement
APT vs. *recA* PCR (54)	0.921	0.055	Almost perfect agreement
MALDI-TOF vs. *recA*Seq^*d*^ (36)	0.942	0.057	Almost perfect agreement
RFLP^*e*^ vs. *recA* Seq (36)	0.592	0.099	Moderate agreement

*a*: number of isolates tested by each test;*b*: adapted interpretation criteria (Landis & Koch 1977);*c*: amplification of *recA*;*d*: sequencing of *recA*;*e*: *Hae*III-restriction fragment length polymorphism (RFLP) *recA*; APT: automated phenotypic tests; CPT: conventional phenotypic tests; k: kappa index; MALDI-TOF: matrix-assisted laser desorption ionization-time of flight; PCR: polymerase chain reaction; SE: standard error.

*RecA* amplification data showed almost perfect agreement with CPT data (k: 0.833) and with APT data (k: 0.921). In addition, MALDI-TOF data was also in almost perfect agreement with *recA* sequencing data (k: 0.942). However, PCR-RFLP and *recA* sequencing data presented moderate agreement (k: 0.592). The phenotypic data and the *recA*amplification data were also compared. CPT and APT had assay specificity of 94.44% and 97.62%, respectively (Table III). Furthermore, the assay sensitivity of CPT and APT were 81.25% and 100%, respectively (Table III).

**TABLE III:** A comparative assessment of automated phenotypic tests (APT) and conventional phenotypic tests (CPT) proficiency for *Burkholderia cepacia* complex (Bcc) identification

Test	Sensitivity (%)	Specificity (%)	PPV (%)	NPV (%)
APT	97.62	100	100	94.12
CPT	94.44	81.25	91.89	86.67

analysis performed with 54 isolates presumptively identified as Bcc. NPV: negative predictive value; PPV: positive predictive value.

PCR-RFLP analysis of 36 isolates resulted in nine different restriction profiles. Three different patterns (B, C, and F) were obtained for *B. cenocepacia* IIIB and one for *B. cenocepacia* IIIA (pattern A). Pattern D was associated with *B. multivorans*. The pattern E was linked with *B. cepacia*. Two patterns (H and I) were related to *B. vietnamiensis*. The pattern G corresponded to C12*B. stabilis* control.

Our data showed prevalence rate of 33.4% (12/36) for *B. cenocepacia*IIIA, 30.6% (11/36) for *B. vietnamiensis*, 27.8% (10/36) for*B. cenocepacia* IIIB, 5.5% (2/36) for *B. multivorans*, and 2.7% (1/36) for *B. cepacia*.

Antimicrobial susceptibility testing data presented as follows: the *B. cepacia* isolate was susceptible to all antimicrobial agents tested; all*B. cenocepacia* (22/22) were susceptible to CAZ, LEV, and MER, and 68.2% (15/22) were susceptible to SUT; all *B. multi-vorans*(2/2) showed sensitivity to LEV, CAZ, and MER, but presented resistance to SUT; all*B. vietnamiensis* (11/11) were sensitive to LEV, 81.8% (9/11)*B. vietna- miensis* were sensitive to CAZ, 90.9% (10/11)*B. vietnamiensis* isolates were sensitive to MER, and 72.7% (8/11) *B. vietnamiensis* isolates were sensitive to SUT.

## DISCUSSION

Owing to the similarity in phenotypic characteristics of Bcc species, interspecies distinction is challenging (Vandamme & Dawyndt 2011). A comparative analysis between CPT and APT was performed using k test and the results indicated that the findings were in agreement; however, it is important to note that the quality of the CPT results depends on the experience and expertise of the microbiologist. A comparative analysis of phenotypic test data and*recA* amplification data indicated that CPT and APT data were in agreement with *recA* amplification data; however, the positive predictive value, specificity, and the k coefficient of APT was higher than that of CPT. Thus, APT may be a better test than CPT for Bcc identification. Although APT cannot adequately perform Bcc interspecies distinction, it could be used for Bcc identification in laboratories lacking molecular biology facilities.

MALDI-TOF proved to be a useful tool for identification of Bcc species obtained from CF patients, although it was not able to identify *B. cenocepacia*subtypes. This finding corroborated those from other studies (Bittar & Rolain 2010, Fehlberg et al. 2013). MALDI-TOF is a rapid and single step assay that does not require personnel with assay expertise. Furthermore, MALDI-TOF and*recA* sequencing assays have comparable Bcc identification accuracy (Bittar & Rolain 2010, Fehlberg et al. 2013).

Studies have shown that *recA* PCR using species-specific primers is not sensitive and specific (Dalmastri et al. 2005, Mahenthiralingam et al. 2008, Vandamme & Dawyndt 2011) and sequencing is required for the final identification (Mahenthiralingam et al. 2002, Vermis et al. 2002a). Comparison between PCR-RFLP and *recA* sequencing data indicates only moderate correlation between the findings of the two methods. Other studies have shown that*Hae*III RFLP patterns insufficiently discriminated *B. cepacia*, *B. cenocepacia*, and *B. stabilis*(Vanlaere et al. 2009, Navrátilová et al. 2013). Previous studies demonstrated the limited usefulness of PCR-RFLP technique for discriminating Bcc: the same variants may have different genomic restriction patterns, one restriction pattern can be associated with different species, and interlaboratory reproducibility is impaired because more than 70*recA*-*Hae*III restriction patterns have been identified. In addition, the requirement of a standard control for each species and its subtypes become the precise identification of each isolate not practical (Vanlaere et al. 2008, Vandamme & Dawyndt 2011). Although the results suggest the optimal performance of MALDI-TOF of Bcc species identification, some studies indicate a certain difficulty of this method to differentiate some strains of*B. cepacia* and *B. cenocepacia* (Vandamme & Dawyndt 2011, Navrátilová et al. 2013).

This study found the highest prevalence rates for *B. cenocepacia* and this finding was similar to those reported in epidemiological studies of patients with CF and Bcc (Mahenthiralingam et al. 2002, Drevinek & Mahenthiralingam 2010, LiPuma 2010). The prevalence of *B. cenocepacia* IIIA determined in our study was similar to that reported in Canada, UK, Italy, and other European countries, while IIIB is prevalent in United States of America (USA) (Mahenthiralingam et al. 2002). However, higher *B. multivorans* prevalence in CF centres in the UK and the USA has been recently reported and the increased prevalence may be due to the approaches taken in these centres to control *B. cenocepacia* outbreaks (Mahenthiralingam et al. 2008, Drevinek & Mahenthiralingam 2010, LiPuma 2010). *B. vietnamiensis* was identified as the second most prevalent genomic variant and these findings were in agreement with those of a previous study (LiPuma et al. 2001).

Antimicrobial susceptibility data showed that the isolates were highly resistant to SUT. Amongst the isolated Bcc species, *B. vietnamiensis* showed a higher percentage of resistance to all antibiotics than that displayed by the other species identified in our study and these findings contradicted previous findings (Vermis et al. 2003), which showed that*B. vietnamiensis* was the most sensitive species among the 142 clinical and environmental Bcc samples. Prolonged and aggressive antibiotic therapy is required for treatment of patients with Bcc chronic infection (Leitão et al. 2008). Therefore, antibiotic susceptibility pattern data for local Bcc strains are important because these microorganisms have intrinsic resistance and develop in vivo resistance to several classes of antimicrobials and thereby limit the effectiveness of empirical antibiotic therapy (Drevinek & Mahenthiralingam 2010). In conclusion, a polyphasic approach that combines biochemical and molecular tests is required to accurate identification of Bcc species infecting CF patients. In the studied population, *B. cenocepacia* was the prevalent species, predominantly IIIA subtype. Identification by MALDI-TOF and sequencing of *recA* agreed in 97.2% of the isolates. Although MALDI-TOF results completely agree with the results of *recA*sequencing for the majority of the strains, it proved not to be able to identify*B. cenocepacia* subtypes. Between the two phenotypic assays, APT was determined to be a better analytical method than CPT was and can be used in laboratories without molecular assay facilities. However, phenotypic assays were not useful for Bcc species differentiation. *B. vietnamiensis* was the Bcc species resistant to the higher number of antimicrobials. SUT was the antimicrobial with the highest percentage of resistance. The high degree of antimicrobial susceptibility obtained in our study may be due the fact that the studied strains are from outpatient, possibly undergoing minor antimicrobial selective pressure.

## References

[B1] Bittar F, Rolain J (2010). Detection and accurate identification of new or emerging
bacteria in cystic fibrosis patients. Clin Microbiol Infect.

[B2] CFC - Cystic Fibrosis Canada (2015). The Canadian cystic fibrosis registry - 2013 annual
report.

[B3] CLSI - Clinical and Laboratory Standards Institute (2012). Methods for dilution antimicrobial susceptibility tests for
bacteria that grow aerobically. Approved standard, CLSI document M7-A9,.

[B4] CLSI - Clinical and Laboratory Standards Institute (2014). Performance standards for antimicrobial susceptibility testing,
Twenty-Fourth Informational Supplement, CLSI document M100-S24.

[B5] Coenye T, Vandamme P, Govan JR, LiPuma JJ (2001). Taxonomy and identification of the Burkholderia cepacia
complex. J Clin Microbiol.

[B6] Dalmastri C, Pirone L, Tabacchioni S, Bevivino A, Chiarini L (2005). Efficacy of species-specific recA PCR tests in the
identification of Burkholderia cepacia complex environmental
isolates. FEMS Microbiol Lett.

[B7] De Boeck K, Malfroot A, Van Schil L, Lebecque P, Knoop C, Govan JRW, Doherty C, Laevens S, Vandamme P (2004). Epidemiology of Burkholderia cepacia complex
colonisation in cystic fibrosis patients. Eur Respir J.

[B8] Drevinek P, Mahenthiralingam E (2010). Burkholderia cenocepacia in cystic fibrosis:
epidemiology and molecular mechanisms of virulence. Clin Microbiol Infect.

[B9] Fehlberg LCC, Andrade LHS, Assis DM, Pereira RHV, Gales AC, Marques EA (2013). Performance of MALDI-ToF MS for species identification
of Burkholderia cepacia complex clinical isolates. Diagn Microbiol Infect Dis.

[B10] Gilligan P, Kiska D, Appleman M (2006). Cumitech 43, Cystic fibrosis microbiology.

[B11] Henry DA, Campbell ME, LiPuma JJ, Speert DP (1997). Identification of Burkholderia cepacia isolates from
patients with cystic fibrosis and use of a simple new selective
medium. J Clin Microbiol.

[B12] Khot PD, Couturier MR, Wilson A, Croft A, Fisher MA (2012). Optimization of matrix-assisted laser desorption
ionization-time of flight mass spectrometry analysis for bacterial
identification. J Clin Microbiol.

[B13] Landis JR, Koch GG (1977). The measurement of observer agreement for categorical
data. Biometrics.

[B14] Leitão JH, Sousa SA, Cunha MV, Salgado MJ, Melo-Cristino J, Barreto MC, Sá-Correia I (2008). Variation of the antimicrobial susceptibility profiles
of Burkholderia cepacia complex clonal isolates obtained from chronically infected
cystic fibrosis patients: a five-year survey in the major Portuguese treatment
center. Eur J Clin Microbiol Infect Dis.

[B15] LiPuma JJ (2010). The changing microbial epidemiology in cystic
fibrosis. Clin Microbiol Rev.

[B16] LiPuma JJ, Spilker T, Gill LH, Campbell PW, Liu L, Mahenthiralingam E (2001). Disproportionate distribution of Burkholderia cepacia
complex species and transmissibility markers in cystic fibrosis. Am J Respir Crit Care Med.

[B17] Mahenthiralingam E, Baldwin A, Dowson CG (2008). Burkholderia cepacia complex bacteria: opportunistic
pathogens with important natural biology. J Appl Microbiol.

[B18] Mahenthiralingam E, Baldwin A, Vandamme P (2002). Burkholderia cepacia complex infection in patients with
cystic fibrosis. J Med Microbiol.

[B19] Mahenthiralingam E, Bischof J, Byrne S, Radomski C, Davies J, Av-gay Y (2000). DNA-based diagnostic approaches for identification of
Burkholderia cepacia complex, Burkholderia vietnamiensis, Burkhol- deria cepacia
genomovars I and III. J Clin Microbiol.

[B20] Navrátilová L, Chromá M, Hanulík V, Raclavský V (2013). Possibilities in identification of genomic species of
Burkholderia cepacia complex by PCR and RFLP. Pol J Microbiol.

[B21] Peeters C, Zlosnik JEA, Spilker T, Hird TJ, LiPuma JJ, Vandamme P (2013). Burkholderia pseudomultivorans sp. nov., a novel Bur-
kholderia cepacia complex species from human respiratory samples and the
rhizosphere. Syst Appl Microbiol.

[B22] Schreckenberger P, Daneshvar M, Weyant R, Hollis D, Murray P, Baron R, Jorgensen J, Pfaller M, (eds.) R Yolken (2003). Acinetobacter, Achromobacter, Chryseobacterium,
Moraxella and other nonfermentative Gram-negative rods. Manual of clinical microbiology.

[B23] Smet B, Mayo M, Peeters C, Zlosnik JE, Spilker T, Hird TJ, LiPuma JJ, Kidd TJ, Kaestli M, Ginther JL, Wagner DM, Keim P, Bell SC, Jacobs JA, Currie BJ, Vandamme PA (2015). Burkholderia stagnalis sp. nov. and Burkholderia
territorii sp. nov., two novel Burkholderia cepacia complex species from
environmental and human sources. Int J Syst Evol Microbiol.

[B24] Souza HAPHM (2012). Detecção precoce e monitoração da colonização por pseudomonas
aeruginosa em crianças com fibrose cística.

[B25] Souza HAPHM, Nogueira KS, Matos AP, Vieira RP, Riedi CA, Rosário NA, Telles FQ, Dalla-Costa LM (2006). Early microbial colonization of cystic fibrosis patients
identified by neonatal screening with emphasis on Staphylococcus
aureus. J Pediatr (Rio J).

[B26] Vandamme P, Dawyndt P (2011). Classification and identification of the Burkholderia
cepacia complex: past, present, and future. Syst Appl Microbiol.

[B27] Vanlaere E, Baldwin A, Gevers D, Henry D, de Brandt E, LiPuma JJ, Mahenthiralingam E, Speert DP, Dowson C, Vandamme P (2009). Taxon K, a complex within the Burkholderia cepacia
complex, comprises at least two novel species, Burkholderia contaminans sp. nov.
and Burkholderia lata sp. nov. Int J Syst Evol Microbiol.

[B28] Vanlaere E, LiPuma JJ, Baldwin A, Henry D, de Brandt E, Mahenthiralingam E, Speert D, Dowson C, Vandamme P (2008). Burkhol- deria latens sp. nov., Burkholderia diffusa sp.
nov., Burkholderia arboris sp. nov., Burkholderia seminalis sp. nov., and Burkhol-
deria metallica sp. nov., novel species within the Burkholderia cepacia
complex. Int J Syst Evol Microbiol.

[B29] Vermis K, Coenye T, Mahenthiralingam E, Nelis HJ, Vandamme P (2002a). Evaluation of species-specific recA-based PCR tests for
genomovar level identification within the Burkholderia cepacia
complex. J Med Microbiol.

[B30] Vermis K, Vandamme P, Nelis HJ (2003). Burkholderia cepacia complex genomovars: utilization of
carbon sources, susceptibility to antimicrobial agents and growth on selective
media. J Appl Microbiol.

[B31] Vermis K, Vandekerckhove C, Nelis HJ, Vandamme PAR (2002b). Evaluation of restriction fragment length polymorphism
analysis of 16S rDNA as a tool for genomovar characterisation within the
Burkholderia cepacia complex. FEMS Microbiol Lett.

